# Amyloid-β prediction machine learning model using source-based morphometry across neurocognitive disorders

**DOI:** 10.1038/s41598-024-58223-3

**Published:** 2024-04-01

**Authors:** Yuki Momota, Shogyoku Bun, Jinichi Hirano, Kei Kamiya, Ryo Ueda, Yu Iwabuchi, Keisuke Takahata, Yasuharu Yamamoto, Toshiki Tezuka, Masahito Kubota, Morinobu Seki, Ryo Shikimoto, Yu Mimura, Taishiro Kishimoto, Hajime Tabuchi, Masahiro Jinzaki, Daisuke Ito, Masaru Mimura

**Affiliations:** 1 Department of Neuropsychiatry, Keio University School of Medicine, 35 Shinanomachi, Shinjuku-Ku, Tokyo, 160-8582 Japan; 2 Office of Radiation Technology, Keio University Hospital, 35 Shinanomachi, Shinjuku-Ku, Tokyo, 160-8582 Japan; 3 Department of Radiology, Keio University School of Medicine, 35 Shinanomachi, Shinjuku-Ku, Tokyo, 160-8582 Japan; 4 Department of Functional Brain Imaging Research, Institute for Quantum Medical Science, National Institutes for Quantum Science and Technology, 4-9-1 Anagawa, Inage-Ku, Chiba-Shi, Chiba, 263-8555 Japan; 5 Department of Neurology, Keio University School of Medicine, 35 Shinanomachi, Shinjuku-Ku, Tokyo, 160-8582 Japan; 6 Department of Physiology, Keio University School of Medicine, 35 Shinanomachi, Shinjuku-Ku, Tokyo, 160-8582 Japan; 7 Memory Center, Keio University School of Medicine, 35 Shinanomachi, Shinjuku-Ku, Tokyo, 160-8582 Japan; 8 Psychiatry Department, Donald and Barbara Zucker School of Medicine, Hempstead, NY 11549 USA; 9 Hills Joint Research Laboratory for Future Preventive Medicine and Wellness, Keio University School of Medicine, Mori JP Tower F7, 1-3-1 Azabudai, Minato-ku, Tokyo, 106-0041 Japan; 10 Center for Preventive Medicine, Keio University, Mori JP Tower 7th Floor, 1-3-1 Azabudai, Minato-ku, Tokyo, 106-0041 Japan

**Keywords:** Alzheimer’s disease, Amyloid-β, Machine learning, Magnetic resonance imaging, Source-based morphometry, Brain imaging, Neurodegenerative diseases, Alzheimer's disease, Dementia

## Abstract

Previous studies have developed and explored magnetic resonance imaging (MRI)-based machine learning models for predicting Alzheimer’s disease (AD). However, limited research has focused on models incorporating diverse patient populations. This study aimed to build a clinically useful prediction model for amyloid-beta (Aβ) deposition using source-based morphometry, using a data-driven algorithm based on independent component analyses. Additionally, we assessed how the predictive accuracies varied with the feature combinations. Data from 118 participants clinically diagnosed with various conditions such as AD, mild cognitive impairment, frontotemporal lobar degeneration, corticobasal syndrome, progressive supranuclear palsy, and psychiatric disorders, as well as healthy controls were used for the development of the model. We used structural MR images, cognitive test results, and apolipoprotein E status for feature selection. Three-dimensional T1-weighted images were preprocessed into voxel-based gray matter images and then subjected to source-based morphometry. We used a support vector machine as a classifier. We applied SHapley Additive exPlanations, a game-theoretical approach, to ensure model accountability. The final model that was based on MR-images, cognitive test results, and apolipoprotein E status yielded 89.8% accuracy and a receiver operating characteristic curve of 0.888. The model based on MR-images alone showed 84.7% accuracy. Aβ-positivity was correctly detected in non-AD patients. One of the seven independent components derived from source-based morphometry was considered to represent an AD-related gray matter volume pattern and showed the strongest impact on the model output. Aβ-positivity across neurological and psychiatric disorders was predicted with moderate-to-high accuracy and was associated with a probable AD-related gray matter volume pattern. An MRI-based data-driven machine learning approach can be beneficial as a diagnostic aid.

## Introduction

Alzheimer’s disease (AD) is a neurodegenerative disorder characterized by the presence of amyloid-beta (Aβ) plaques, neurofibrillary tangles, and brain atrophy^1,2^. It is the most prevalent cause of dementia^3–5^ and has a significant social impact^4^. However, the clinical diagnosis of AD can be challenging due to overlapping clinical manifestations with other diseases such as frontotemporal lobar degeneration (FTLD) or late-onset psychiatric disorders. These diseases may present similar clinical signs and symptoms and occasionally may be comorbid with AD^3,6,7^.

Considering that Aβ is one of the defining characteristics of AD, examining Aβ-positivity may aid in differential diagnosis^2,8^ and precision medicine, including drug choice^6,9^. Nevertheless, Aβ detection is not necessarily convenient to perform in routine clinical practice. Positron emission tomography (PET)^10,11^ requires advanced facility requirements and careful attention to radiation exposure. Cerebrospinal fluid (CSF) testing^12,13^ can be risky in patients with bleeding tendencies (e.g., on anticoagulants) or increased intracranial pressure. Blood biomarkers have shown potential for high diagnostic performance in a minimally invasive manner^14–16^, but have not been applied in routine clinical practice^17^. Meanwhile, magnetic resonance imaging (MRI) has achieved widespread adoption in general clinical practice despite certain facility limitations. As MRI may be effective in excluding non-AD causes of cognitive impairment and contribute to the diagnosis of dementia^17^, MRI-based Aβ prediction may be a useful screening tool before definitive diagnosis by CSF testing or amyloid PET^18–20^.

However, visual judgment of MRI may be hindered by the heterogeneity of brain structural changes. In other words, objective, data-driven detection of subtle structural changes indicative of Aβ deposition can enhance the visual interpretation of MRI for dementia differential diagnosis, streamlining the screening process for potential participants undergoing CSF testing or amyloid PET scans^18–22^.

Large MRI datasets such as Alzheimer’s Disease Neuroimaging Initiative (ADNI)^23^ has facilitated data-driven approaches (e.g., machine learning) (for reviews, see Refs.^21,22^). Machine learning methods utilizing these datasets have achieved classification accuracy of 93–98% in distinguishing between AD and healthy controls (HC)^24,25^. However, one of the limitations of the previous studies is the narrow focus on the AD continuum, including AD, mild cognitive impairment (MCI), and HC^20,24–27^. Consequently, the results may not necessarily be generalizable to common clinical populations^6,18,28,29^. Another limitation of previous MRI-based models is that many predict clinical diagnoses^24,25^ (for reviews, see Refs.^21,22^) instead of Aβ deposition^18,26–28^.

To examine the brain structure patterns in neurological or psychiatric disorders, source-based morphometry (SBM), a data-driven multivariate analysis method, has garnered increasing attention^30–35^. SBM is a structural neuroimaging analysis technique based on independent component analysis (ICA). It uses masked gray matter (GM) images as input features, extracts independent spatial maps representing anatomical variability, and potentially detects co-varying structural patterns in the whole brain^30,35^. In this regard, SBM may be more data-driven than region-of-interest (ROI)-based feature extraction and potentially more sensitive than voxel-based morphometry for detecting GM atrophy^30^. SBM has significantly contributed to enhancing our comprehension of distinctive brain structural patterns observed in patients affected by various neurological or psychiatric disorders, including Parkinson's disease^31^, FTLD syndrome^33^, major depressive disorder^34^, and schizophrenia^32^. Applying SBM to the AD continuum, the temporo-frontoparietal component could differentiate amnestic MCI (aMCI) from HC,major hippocampal and temporal lobe atrophy and occipital atrophy could differentiate AD from aMCI and HC^31^.

Considering the challenges posed by the presence of AD-like signs and symptoms in other neurological or psychiatric disorders, which likely hamper diagnosis and patient management, predicting Aβ accumulation based on MR-images of heterogeneous diseases may hold greater clinical relevance. The aims of the present study were to (1) build a clinically useful prediction model for Aβ deposition from a diverse patient population using SBM, and (2) identify influential features in the model. Moreover, we assessed how the predictive accuracies varied with feature combinations.

## Methods

### Participants and clinical measurements

Patients clinically diagnosed with AD, MCI, FTLD, corticobasal syndrome (CBS), progressive supranuclear palsy (PSP), or psychiatric disorders were recruited between the 3^rd^ of July, 2018 and 31^st^ of August, 2021, from the memory clinic at Keio University Hospital. HC were also recruited as described in a previous manuscript^36^. For these diagnoses, the PET results were not considered.

Inclusion criteria among each diagnosis were: (1) age 40–85 years; (2) years of education ≥ 12, and (3) patients whose first language is Japanese.

The diagnosis-specific inclusion criteria were as follows:

AD: (1) clinical diagnosis of AD by a dementia specialist; (2) Logical Memory II subscale in the Wechsler Memory Scale-Revised (LM II) ≤ 8 for 16 years of education and ≤ 4 for 12–15 years of education; (3) Mini-Mental Scale Examination (MMSE) ≤ 23; and (4) clinical dementia rating (CDR) = 0.5 or 1.0.

MCI: (1) Clinical diagnosis of MCI by a dementia specialist; (2) LM II ≤ 11 for 16 years of education, and ≤ 9 for 12–15 years of education; (3) MMSE ≥ 24; and (4) CDR 0.5 (memory score 0.5).

Other diseases (i.e., FTLD, CBS, PSP, or psychiatric disorders) were diagnosed by a neurologist or psychiatrist according to the diagnostic criteria.

HC: (1) judged as cognitively normal by a dementia specialist; (2) LM II ≥ 9 for 16 years of education, ≥ 5 for 12–15 years of education; (3) MMSE ≥ 24; (4) CDR 0; and (5) Geriatric Depression Scale (GDS) < 6.

All the clinical data were obtained within 6 months from enrollment.

### Standard protocol approval, registration, and patient consent

The Certified Review Board of Keio University approved the study design and protocol. The study was registered with the University Hospital Medical Information Network Clinical Trials Registry (UMIN-CTR; https://www.umin.ac.jp/ctr/index.htm, ID# UMIN000032027, the first registration: 31/03/2018) and the Japan Registry of Clinical Trials (jRCT; https://jrct.niph.go.jp/, ID# jRCTs031180225, the first registration: 11/03/2019), and was conducted in accordance with the 1964 Declaration of Helsinki and its later amendments. All participants and their proxies, if necessary, provided written informed consent.

### Cognitive assessment

The following neuropsychological assessments were performed: MMSE^37^, Wechsler Memory Scale-Revised (WMS-R) Logical Memory^38^ immediate recall (LM I) and delayed recall (LM II), Word Fluency^39^, Trail Making Test (TMT)^40^, the Japanese version of Alzheimer’s Disease Assessment Scale-Cognitive subscale (ADAS-cog-J)^41^, Japanese Adult Reading Test (JART)^42^, Clinical Dementia Rating (CDR)^43^, and Functional Activity Questionnaire (FAQ)^44^.

### Apolipoprotein E (APOE) genotyping

Genomic DNA was extracted from 0.2 mL whole blood using a Magnetic Nanoparticle DNA Extraction Kit (EZ1 DNA Blood 200 μL Kit). APOE genotyping (rs429358 and rs7412) was performed by real-time polymerase chain reaction (PCR) using the TaqMan probe on a CFX 96 deep well Real-Time PCR system (Bio Rad, Richmond, CA) to analyze the three major isoforms (APOE ε2, ε3, and ε4).

### [^18^F] Florbetaben (FBB) amyloid-PET imaging

[^18^F] FBB was manufactured on-site using an automated synthesizer as described elsewhere^45,46^. Amyloid-PET images were acquired for 20 min using a PET-CT (True Point Biograph 40/64, Siemens Japan K.K., Tokyo, Japan), 90 min after intravenous injection of 300 MBq ± 20% [^18^F] FBB. The 20-min PET images were visually assessed by nuclear medicine experts who had completed a training program offered by the manufacturer (Piramal Imaging GmbH, Berlin, Germany). The Aβ positivity/negativity was determined based on the assessment of tracer uptake in the GM of the following four brain regions: the lateral temporal lobes, frontal lobes, posterior cingulate cortex/precuneus, and parietal lobes, in line with the NeuraCeq™ guidelines (http://www.accessdata.fda.gov/drugsatfda_docs/label/2014/204677s000lbl.pdf)^47^. Aβ negativity was established when tracer uptake (i.e., signal intensity) in the GM was lower than that in the white matter (WM) in all four brain regions.

### MRI acquisition

High-resolution 3D T1-weighted MR-images were acquired (repetition time: 6.8 ms; echo time: 3.0 ms; flip angle: 8°; field of view: 230 mm; matrix size: 256 × 256; slice thickness: 1.0 mm; voxel size: 0.9 × 0.9 × 1.0 mm) using a Discovery MR750 3.0 T scanner (GE Healthcare, USA) with a 32-channel head coil. All images were visually checked for scanner artifacts and anatomical anomalies.

### MRI pre-processing

Structural brain images were first segmented into GM, WM, and CSF using the Statistical Parametric Mapping (SPM12; Wellcome Trust Center for Neuroimaging, London, UK) toolbox CAT12 (http://www.neuro.uni-jena.de/cat/) in MATLAB (R2019a; MathWorks, Natick, Mass, USA). Segmented GM images were used to normalize the individual component images to the Montreal Neurological Institute (MNI) template^48^. Normalized images were modulated to preserve the total amount of signal from each voxel, resampled to an isotropic voxel size of 2 × 2 × 2 mm^3^, and smoothed using a 5-mm full-width-at-half-maximum Gaussian kernel.

For the subsequent pre-processing, we used SBM^30,35^. SBM incorporates independent component analysis (ICA) and provides automatic decomposition of a given set of anatomical brain images into independent spatial maps characterizing different modes of anatomical variability across all individuals^30,35^.

The preprocessed GM images were loaded with Nibabel (https://nipy.org), and a three-dimensional (3D) array of 91 × 109 × 91 voxels was transformed into a one-dimensional (1D) array of 1 × 902,629 voxels. We created a brain mask for this 1D array using the Neuromorphometric Atlas (provided by Neuromorphometrics, Inc. (http://Neuromorphometrics.com))^49^ and selected 208,082 voxels on which ICA was performed for all scans using the FastICA function of scikit-learn (https://scikit-learn.org/stable/), a Python machine learning library. The number of extracted independent components (ICs) was also used as a definitive hyperparameter to be tuned in subsequent model building.

After conducting the ICA, we reshaped the data matrix (i.e., ICs) back into a 3D image (91 × 109 × 91) using nipy (https://nipy.org). The 3D image was then superimposed onto the MNI-normalized template brain using BrainNet Viewer^50^, for visualization. The extracted ICs were used as spatial regressors for each participant's GM images (I_GM_).

$${\text{I}}_{{{\text{GM}}}} = \, {\rm \beta_{{1}}} {\text{IC}}_{{1}} + \, {\rm \beta_{{2}}} {\text{IC}}_{{2}} + ...{\rm \beta_{{\text{K}}}} {\text{IC}}_{{\text{K}}} .$$

In the above formula, each β represents the weighting coefficient associated with the effect of each IC for the GM image and K indicates the number of extracted ICs. Accordingly, the β-values could be loosely regarded as “weighted total gray matter volume” of the brain parcel represented by the given IC^51^. The β-values were then used as representative GM measures associated with each component, in the subsequent analyses.

### Machine learning

We built predictive models for Aβ-positivity using scikit-learn (https://scikit-learn.org/stable/index.html)^52^ which is supported by Python ver. 3.4. The input feature values were based on the ICA’s β-values, demographic characteristics (i.e., age and sex), cognitive assessments, and APOE genotype. First, we used all input features and built the final model. Second, we investigated the model performance for each combination of features (e.g., brain images alone, brain images and cognitive assessments). Third, we investigated model performance for each combination of diagnoses (e.g., AD + HC and AD + MCI + HC).

Throughout the model building, we used a Gaussian kernel support vector machine (SVM) as the classifier and the model was validated using fivefold cross-validation (Additional Fig. 1). For a fivefold training/test split, the model was fitted to the training data, and the predictive value was assessed using the test data over all splits (five times). We tuned the hyperparameters (i.e., Gamma and C in SVM and the number of ICs) with a grid search in all model buildings.

To improve the interpretability of the model, we applied the SHapley Additive exPlanations (SHAP) (https://shap.readthedocs.io/en/latest/index.html) which makes the output of any machine learning model explainable as a model itself^53^. Based on the Shapley value in game theory, a large absolute SHAP value has a strong influence on the prediction. In the present study, the clinical features with positive and negative SHAP values were associated with Aβ-positivity and Aβ-negativity, respectively.

### Statistical analysis

For the statistical analyses, we used Scipy (https://www.scipy.org), supported by Python version 3.4. Demographic and clinical variables were compared using a two-tailed t-test, or chi-square test, where appropriate. Relationships among features were examined using Pearson’s correlation analysis for continuous variables. Analysis of variance (ANOVA) was conducted to determine associations with diagnoses. Statistical significance was defined by a p-value of < 0.01 or < 0.05 after the Bonferroni correction for multiple corrections.

### Ethics approval and consent to participate

The study protocol was prepared in accordance with the ethical standards of the Declaration of Helsinki and approved by the Certified Review Board of Keio University. Written informed consent was obtained from all participants who were included in the study and their proxies, if necessary.

## Results

### Demographic and clinical characteristics

Among 118 cases used for the final model building (AD [n = 24], MCI [n = 29], FTLD [n = 12], CBS [n = 3], PSP [n = 3], psychiatric disorders [n = 5], HC [n = 42)), 45 cases (38.1%) were Aβ-positive and 73 cases (61.9%) were Aβ-negative (Table 1). The demographic and clinical characteristics are shown in Table 1.

**Table 1:** Demographic and clinical characteristics.

	Aβ-positive (n = 45)		Aβ-negative (n = 73)		t value	p-value
	Mean ± SD		Mean ± SD			
Demographics^a^						
Age (years)	72.38 ± 9.09		67.71 ± 9.78		2.58	0.011
Education (years)	14.47 ± 1.85		14.73 ± 2.29		− 0.64	0.523
Cognitive tests						
MMSE	22.73 ± 5.96		27.37 ± 3.39			
CDR global	0.53 ± 0.33		0.23 ± 0.34			
CDR sum	2.22 ± 2.38		0.84 ± 1.73			
FAQ	4.18 ± 4.44		1.58 ± 3.31			
LM I	4.07 ± 3.58		10.05 ± 5.08			
LM II	2.44 ± 3.58		8.99 ± 5.64			
ADAS-cog-J	14.94 ± 9.99		6.82 ± 6.68			
WF category	27.98 ± 13.29		35.47 ± 12.83			
WF initial	21.53 ± 9.24		24.3 ± 10.92			
TMT-J A	153.58 ± 239.11		83.79 ± 156.68			
TMT-J B	310.22 ± 326.13		131.32 ± 163.87			
JART	25.42 ± 15.21		32.49 ± 13.38			

	N	Men (%)	N	Men (%)	χ^2^	p-value
Diagnosis^b^						
All	45	53.00	73	54.79	51.09	< 0.01*
AD	21	52.38	3	33.33	33.46	< 0.01*
MCI	16	56.25	13	61.54	13.41	< 0.01*
FTLD	1	0.00	11	54.55	0.03	
CBS	2	50.00	1	0.00	2.90	
PSP	0	0.00	3	33.33	0.10	
Psychiatric	0	0.00	5	40.00	0.00	
HC	5	60.00	37	59.46	–	
APOE allele^b^						
ε2/2	0	0.00	2	0.00	NaN	NaN
ε2/3	0	0.00	8	62.50		
ε2/4	0	0.00	0	0.00		
ε3/3	21	61.90	47	53.19		
ε3/4	18	38.89	16	62.50		
ε4/4	6	66.67	0	0.00		

Values are expressed as mean ± SD unless otherwise indicated. The between-group differences were examined using the independent sample t-test (a) for continuous variables, and χ^2^ test (b) for categorical variables.

*AD* Alzheimer’s disease, *ADAS-cog-J* the Japanese version of Alzheimer’s Disease Assessment Scale-Cognitive subscale, *APOE* Apolipoprotein E, *CBS* Corticobasal syndrome, *CDR* Clinical Dementia Rating, *FAQ* Functional Activity Questionnaire, *FTLD* Frontotemporal lobar degeneration, *HC* Healthy controls, *JART* Japanese Adult Reading Test, *LM I* Logical Memory immediate recall, *LM II* Logical Memory delayed recall, *MCI* Mild cognitive impairment, *MMSE* Mini-Mental State Examination, *PSP* Progressive supranuclear palsy, Psychiatric Psychiatric disorders, *SD* Standard deviation, *TMT-J* The Japanese version of Trail Making Test, *WF* Word Fluency.

*p < 0.01.

### Model performance

**Figure 1:**
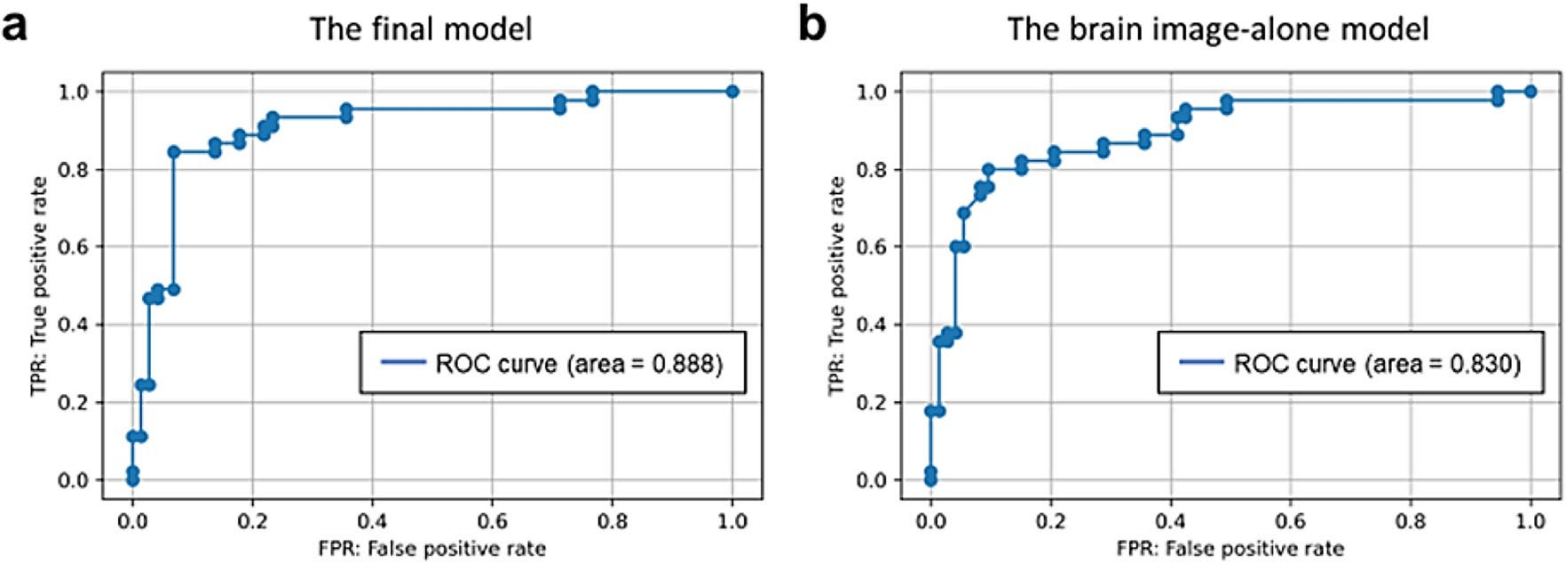
The area under the curve (AUC) of the final model and the brain image-alone model. The area under the receiver operating characteristic curve (AUC) of the final model (**a**) was 0.888 (95% CI 0.854–0.973), and of brain image-alone model (**b**) was 0.830 (95% CI 0.825–0.958).

The final model (C = 0.01, gamma = 100, number of ICs = 7), which used brain images, cognition, and APOE data as input features achieved 89.8% accuracy (sensitivity = 88.4%, specificity = 90.7%, positive predictive value = 84.4%, negative predictive value = 93.2%), whereas the model based on brain images alone showed 84.7% accuracy (sensitivity = 82.9%, specificity = 85.7%, positive predictive value = 75.6%, negative predictive value = 90.4%) (Table 2). The area under the receiver operating characteristic curve (AUC) of the final model was 0.888 (95% confidence interval [CI] 0.854–0.973) and that of the brain images alone model was 0.830 (95% CI 0.825–0.958) (Fig. 1). The final model performance based on the combination of each feature set is presented in Table 2.

**Table 2:** Model performance using brain image, cognition, and APOE data for input features (by feature set).

Feature	Accuracy*^1^	Sensitivity*^2^	Specificity*^3^	Positive predictive value*^4^	Negative predictive value*^5^
	%	%	%	%	%
Brain image	84.7	82.9	85.7	75.6	90.4
Cognition	78.8	71.7	83.3	73.3	82.2
APOE	81.4	74.5	85.9	77.8	83.6
Brain image + Cognition	84.7	84.6	84.8	73.3	91.8
Brain image + APOE	87.3	84.1	89.2	82.2	90.4
ALL	89.8	88.4	90.7	84.4	93.2

*^1^Accuracy = (TP + TN)/(TP + TN + FN + FP).

*^2^Specificity = TN/(FP + TN).

*^3^Sensitivity = TP/(TP + FN).

*^4^Positive predictive value = TP/(TP + FP).

*^5^Negative predictive value = TN/(FN + TN). “ALL” model used brain image (i.e., gray matter volume), cognition, and APOE data.

*APOE* apolipoprotein E., *FN* false negative, *FP* False positive, *TN* True negative, *TP* True positive.

Table 3 shows the performance of the final model to predict Aβ positivity in each diagnosis. The final model achieved an accuracy of 89.8% when including all the participants. The accuracy of the model based on AD, MCI, and HC was slightly lower (i.e. 88.4%), whereas that based solely on MCI was the lowest (i.e. 75.9%). Notably, Aβ-positivity/negativity was completely (i.e. 100%) identified in FTLD syndromes and in psychiatric disorders.

**Table 3:** Model performance using brain image, cognition, and APOE data for input features (by diagnosis set).

Diagnosis	Accuracy*^1^	Sensitivity*^2^	Specificity*^3^	Positive predictive value*^4^	Negative predictive value*^5^
	%	%	%	%	%
HC	92.9	40.0	100.0	100.0	92.5
AD	95.8	95.2	100.0	100.0	75.0
HC + AD	93.9	84.6	100.0	100.0	90.9
MCI	75.9	81.3	69.2	76.5	75.0
HC + MCI	85.9	71.4	92.0	78.9	88.5
HC + MCI + AD	88.4	83.3	92.5	89.7	87.5
FTLD	100.0	100.0	100.0	100.0	100.0
CBS	100.0	100.0	100.0	100.0	100.0
PSP	100.0	N/A	100.0	N/A	100.0
Psychiatric	100.0	N/A	100.0	N/A	100.0
ALL	89.8	88.4	90.7	84.4	93.2

*^1^Accuracy = (TP + TN)/(TP + TN + FN + FP).

*^2^Specificity = TN/(FP + TN).

*^3^Sensitivity = TP/(TP + FN).

*^4^Positive predictive value = TP/(TP + FP).

*^5^Negative predictive value = TN/(FN + TN). The “ALL” model uses data from HC and patients with AD, MCI, FTLD, CBS, PSP, or other psychiatric disorders.

*AD* Alzheimer’s disease, *CBS* Corticobasal syndrome, *FN* False negative, *FP* False positive, *FTLD* Frontotemporal lobar degeneration, *HC* Healthy controls, *MCI* Mild cognitive impairment, *PSP* Progressive supranuclear palsy, *Psychiatric* Psychiatric disorders, *TN* True negative, *TP* True positive.

### SBM

Seven ICs (IC 1–7) were derived from the final SBM model (Table 4 and Additional Fig. 2). Each component showed spatially maximally independent GM volume patterns. Upon examining the relationship between each component and clinical information, IC 1 showed a significant correlation with cognitive measures and Aβ-positivity. Meanwhile, IC 4 was significantly correlated with age (Table 4).

**Table 4:** Relation between each independent component and clinical data.

	IC_1		IC_2		IC_3		IC_4		IC_5		IC_6		IC_7	
	r	p-val	r	p-val	r	p-val	r	p-val	r	p-val	r	p-val	r	p-val
Gender	− 0.036	0.349	− 0.145	0.059	− 0.092	0.160	− 0.019	0.419	0.597	0.000*	0.140	0.066	− 0.300	0.000*
Age	− 0.271	0.002*	− 0.032	0.364	− 0.216	0.009*	− 0.556	0.000*	0.117	0.104	0.244	0.004*	0.194	0.018
Education	0.043	0.321	0.012	0.451	− 0.105	0.128	0.262	0.002*	0.243	0.004*	0.003	0.488	− 0.123	0.092
MMSE	0.498	0.000*	− 0.046	0.312	− 0.186	0.022*	0.120	0.099	0.196	0.017*	0.070	0.226	− 0.354	0.000*
CDR_Global	− 0.371	0.000*	− 0.149	0.054*	0.194	0.017*	− 0.206	0.013*	− 0.140	0.066	− 0.114	0.110	0.315	0.000*
CDR_Sum	− 0.397	0.000*	− 0.102	0.136	0.134	0.074	− 0.168	0.034*	− 0.151	0.051*	− 0.192	0.019*	0.335	0.000*
FAQ	− 0.326	0.000*	− 0.070	0.227	0.166	0.036*	− 0.166	0.036*	− 0.140	0.066	− 0.224	0.007*	0.330	0.000*
LM_I	0.447	0.000*	0.042	0.328	− 0.141	0.064	0.198	0.016*	0.096	0.150	0.083	0.185	− 0.271	0.001*
LM_II	0.461	0.000*	0.063	0.248	− 0.088	0.171	0.221	0.008*	0.046	0.312	0.106	0.126	− 0.246	0.004*
ADAS-cog-J	− 0.461	0.000*	0.007	0.469	0.107	0.124	− 0.168	0.035*	− 0.114	0.110	− 0.165	0.037*	0.487	0.000*
WF_Category	0.374	0.000*	0.083	0.187	− 0.146	0.058	0.232	0.006*	0.122	0.095	0.142	0.063	− 0.325	0.000*
WF_Initial	0.242	0.004*	0.021	0.412	− 0.122	0.094	0.219	0.009*	0.089	0.169	0.173	0.031*	− 0.254	0.003*
TMT-J_A	− 0.329	0.000*	− 0.109	0.120	0.189	0.020*	− 0.060	0.260	− 0.098	0.145	− 0.011	0.451	0.240	0.004*
TMT-J_B	− 0.419	0.000*	− 0.034	0.356	0.218	0.009*	− 0.122	0.094	− 0.144	0.060	− 0.047	0.308	0.325	0.000*
JART	0.261	0.002*	0.005	0.477	− 0.244	0.004*	0.102	0.137	0.278	0.001*	− 0.111	0.115	− 0.239	0.005*
Aβ-positivity	− 0.516	0.000*	− 0.037	0.347	0.224	0.007*	− 0.088	0.172	− 0.090	0.167	0.097	0.148	0.065	0.241

A group comparison analysis of variance (ANOVA) was conducted, and *p < 0.05 after Bonferroni correction.

*ADAS-cog-J* The Japanese version of Alzheimer’s Disease Assessment Scale-Cognitive subscale, *Aβ* amyloid-β, *CDR* Clinical Dementia Rating *FAQ* Functional Activity Questionnaire, *JART* Japanese Adult Reading Test, *LM I* Logical Memory immediate recall, *LM II* Logical Memory delayed recall, *MMSE* Mini-Mental State Examination, *TMT-J* The Japanese version of Trail Making Test, *WF* Word Fluency.

**Figure 2:**
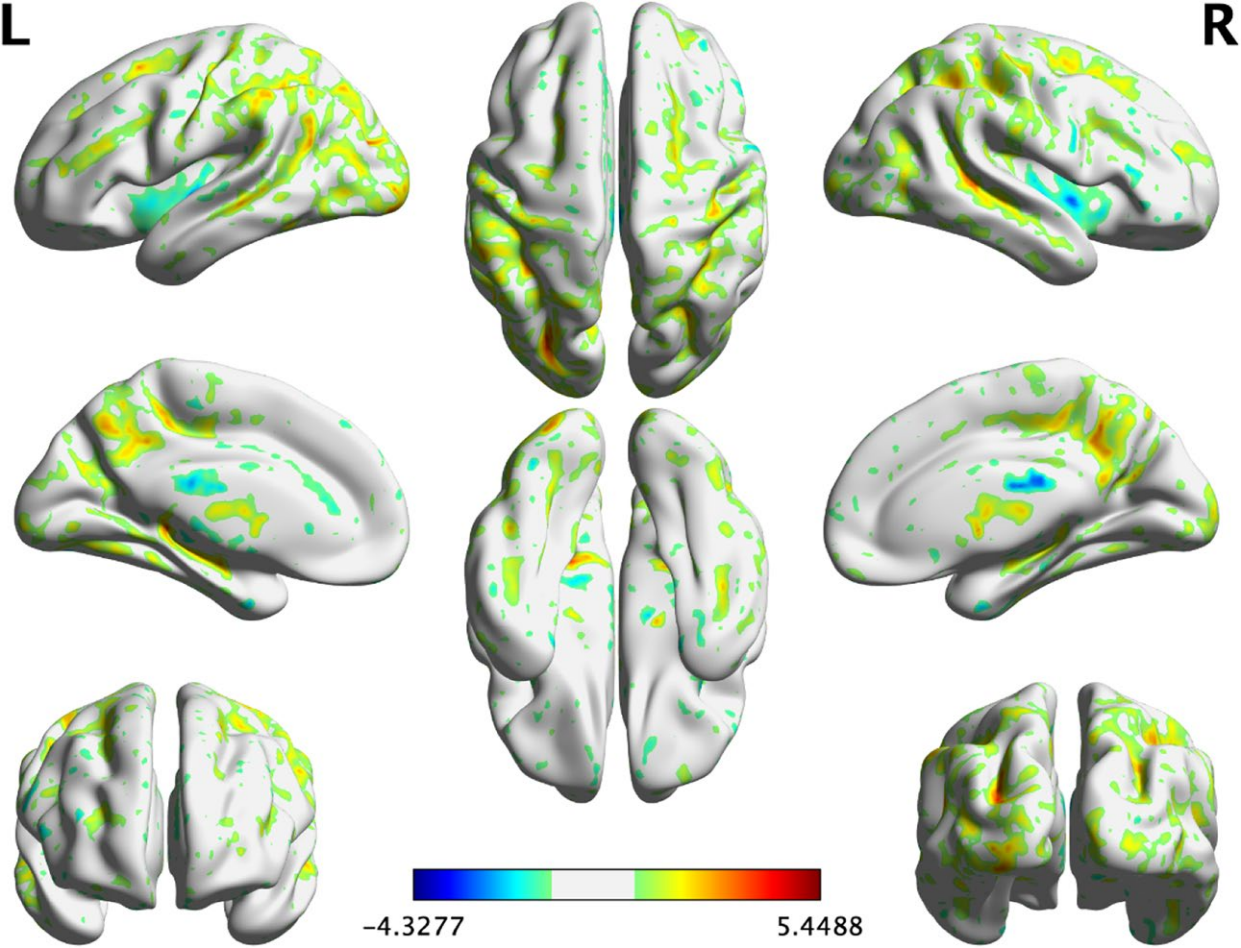
The gray matter volume pattern of independent component 1 in a three-dimensional brain map derived from source-based morphometry. A three-dimensional brain map of independent component 1. The color bar indicates the z-score. The z-score is calculated as (value—mean) / standard deviation, and regions with z-scores greater than or equal to 1 are color-coded. The 3D image was generated using BrainNet Viewer 1.7 (https://www.nitrc.org/projects/bnv).

We assessed whether each clinical diagnosis was associated with the ICs. Only AD-diagnosis and IC 1 showed a significant association (Games-Howell test was applied for multiple comparisons, p < 0.001), whereas the other diagnoses were not associated with any ICs. The GM volume pattern of IC 1 is shown in Fig. 2. The spatial pattern of the loading coefficients from IC 1 showed higher z-scores in the lateral parietal lobes than in the other ICs.

### Feature importance of the model

**Figure 3:**
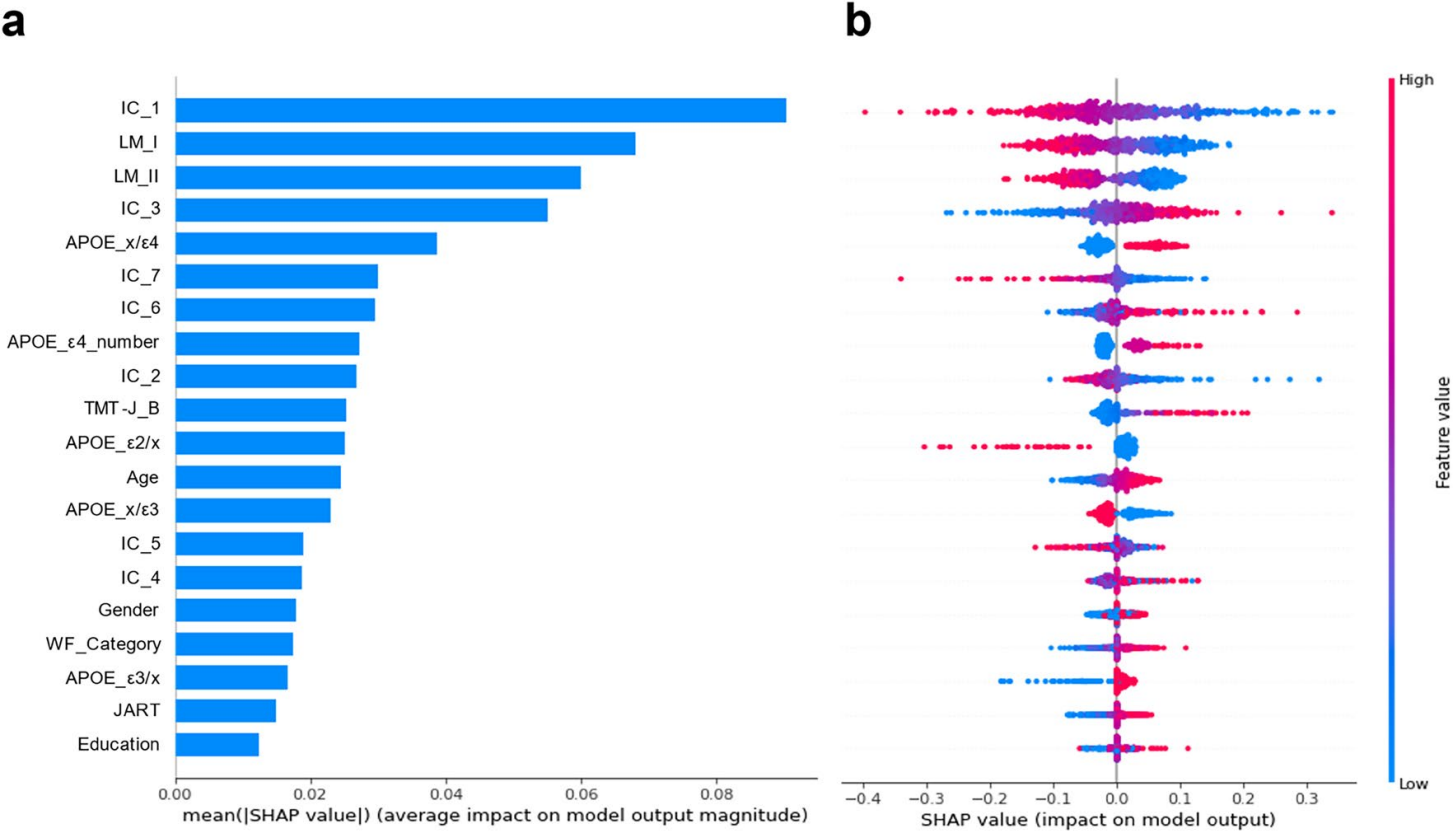
Mean SHAP value in fivefold cross-validation. The horizontal and vertical axes represent the mean SHAP value in fivefold cross-validation and features, respectively. (**a**) Shows the relationship between each feature and the absolute value of SHAP in the analysis. A large absolute SHAP value indicates a significant influence on the prediction. (**b**) Shows the SHAP values for each participant. This plot summarizes how the top features in the dataset affect the output of the model in the form of information density. The x position of the dots is based on the SHAP value of the feature, and the dots are stacked along each feature row to indicate density. Positive and negative SHAP values were associated with Aβ-positivity and Aβ-negativity, respectively. The red dots indicate high values for each feature, while the blue dots indicate low values for each feature. If the red dots are in the positive SHAP, then the higher the feature value, the more it contributes to the Aβ-positivity. Likewise, if blue dots are in the positive SHAP, the lower the feature value, the more it contributes to the Aβ-positivity. For example, lower scores on immediate and delayed recall of Logical Memory (i.e., LM I and LM II) were associated with Aβ-positivity. *IC* independent component, *JART* Japanese Adult Reading Test *LM* Logical Memory, *SHAP* SHapley Additive Explanations, *TMT-J* The Japanese version of Trail Making Test, *WF* Word Fluency.

The SHAP values were calculated (Fig. 3), in which IC 1 showed the strongest impact on the model, followed by Logical Memory I and II, IC 3, and APOE x/4.

## Discussion

Using SBM, our machine learning model predicted Aβ-positivity with an accuracy of 89.8% and an AUC of 0.888 based on brain MRI, cognitive, and genetic data from 118 participants. It also correctly predicted Aβ-positivity/negativity in non-AD participants, such as those with FTLD syndrome and psychiatric disorders. Even a model based solely on brain images achieved 84.7% accuracy and an AUC of 0.830. Among all the covariates in the final model, IC 1 had the strongest impact related to Aβ-positivity prediction, followed by Logical Memory I and II. This suggests that our model may be beneficial in clinical settings.

### Model performance

Our model yielded the best accuracy (i.e. 89.8%) when it included non-AD cases, whereas the model based only on the AD continuum achieved slightly lower accuracy (i.e. 88.4%). It can be interpreted that the heterogeneity of clinical features among non-AD participants was informative in refining the accuracy of the final model.

While numerous machine learning models based on brain images have been developed, most of them have focused on the clinically determined AD continuum^20,24–27^, and predicted the clinical diagnoses of AD instead of imaging/pathology-based Aβ deposition^18,28^.

As patients visiting physicians’ offices would have various neurocognitive disorders beyond the AD continuum^18,26,27^, our model, which was based on diverse clinical populations may be better suited for application in clinical settings. Even our model, based only on structural brain images which yielded an 84.7% accuracy, may assist clinicians’ deciding and screening of potential candidates for AD-related clinical trials. These results may be due to the advantages of SBM, namely its ability to detect subtle morphological changes and unknown patterns in brain structures associated with neurodegenerative diseases without relying on existing atlases^30,35^. These strengths could be exploited in a patient population with diversified diseases, as in this study.

Our model achieved a predictive accuracy of 75.9% for Aβ-positivity in individuals with MCI. Notably, it surpassed the accuracy of the physicians’ clinical diagnosis of AD, which is approximately 70%^3^. Furthermore, our model demonstrated predictive accuracy comparable to previous studies that aimed to predict Aβ-positivity^26^ or future AD diagnosis in MCI patients using structural MRI^20^.

While no definitive treatment is currently available to slow the progression of AD^54^, new drugs aimed at disease-modifying therapies are being approved in some countries^55^. In the context of the growing availability of disease-modifying drugs for AD, accurate and early diagnosis will become a higher priority^55^. Although Aβ deposition is one of the earliest detectable pathological changes in AD^2,6,8,19^, its detection by PET or CSF test may be hampered by the need for specialized facilities, length of time required, or some degree of invasiveness or risk^14–16^. Since MRI is safe and applicable to a wide population, an MRI-based Aβ prediction model based on a heterogeneous population may be valuable for clinicians.

### Feature importance

SHAP analyses indicated that IC 1, LM I, and LM II were important predictive features. These three leading features showed two or more strong impacts compared to the others.

IC 1, the most important feature in our model, was significantly correlated with Aβ-positivity (r = 0.516) and most of the cognitive measures included in the analyses, as shown in Table 4. Furthermore, the spatial pattern of the loading coefficients from IC 1 roughly followed the “cortical pattern” of neurodegeneration in AD that is characterized by cortical atrophy, particularly in the parietal lobe^56^ as depicted in Fig. 1. The parietal lobe, including the precuneus, is known to contribute to episodic memory^57–59^ which is likely to be impaired in AD^60,61^, and is possibly associated with Aβ pathology^62,63^. In our study, however, another “typical AD” pattern^56^, medial temporal lobe (MTL) atrophy^64^, was not observed in any IC. One possibility is that MTL atrophy does not necessarily indicate Aβ pathology, but may be a signal for tau pathology, such as primary age-related tauopathy^65^ or coexistent transactive response DNA-binding protein 43 pathology^66^. These clinicopathological relationships may explain why IC 1 was of greater importance in the prediction and represented the AD-related GM volume pattern.

The importance of Logical Memory scores indicated that memory impairment, a typical cardinal symptom of AD^67^, will also be essential for prediction.

APOE-ε4, a widely-accepted AD risk factor^68^, was also indicated as an important feature, as both “APOE x/ε4” (i.e., ε2/ε4, ε3/ε4, ε4/ε4) and “APOE ε4_number” (i.e., pairwise or not) showed large SHAP values.

Interestingly, all ICs showed greater importance than demographic and cognitive features, including scores on the MMSE, an assessment scale suitable primarily for screening for dementia. Among the ICs, IC 4 was uniquely extracted as a normal aging GM volume pattern (Additional Fig. 3) and lacked any significant association with cognitive measures or Aβ-positivity (Table 4). The separate associations between IC 1 and Aβ-positivity and between IC 4 and age might indicate that our model discriminates AD-related neurodegeneration from normal aging in brain imaging. These results imply that the pathological process of AD is not necessarily age dependent. In other words, brain atrophy patterns in normal aging processes can be distinguished from those in neurodegenerative diseases^51^, even though the deposition of Aβ plaques is likely to increase with age, and several age-related pathologies may be comorbid with AD^69,70^.

Overall, the SHAP analyses imply that SBM-derived GM volume patterns and Logical Memory results might be important for predicting Aβ-positivity across diverse neurocognitive disorders.

## Limitation

This study has some limitations. First, Aβ-positivity was determined only by amyloid-PET scan, whereas CSF Aβ would be a more sensitive marker, particularly in the pre-clinical status^9^. Second, the number of samples in machine learning is expected to affect accuracy^71^, however, our study had a limited number of samples. Therefore, future studies will require larger sample sizes and independent test datasets^72^. Third, longitudinal follow-up data might improve model performance, rather than a cross-sectional approach^73^.

## Conclusions

Our model achieved 89.8% accuracy to predict Aβ-positivity across a diverse range of neurological and psychiatric disorders. Notably, the SBM revealed a GM volume pattern that had the strongest impact on prediction. Even when using structural brain images alone, the accuracy still reached 84.7%. This MRI-based data-driven machine learning approach may aid clinicians in patient management and early decision-making processes.

### Supplementary Information

Supplementary Legends.

Supplementary Figure 1.

Supplementary Figure 2.

Supplementary Figure 3.

## Data Availability

The data and code supporting the conclusions of this article are available from the corresponding author or J.H (hjinichi@keio.jp), upon reasonable request.

## Abbreviations

AD: Alzheimer’s disease
ADAS-cog-J: The Japanese version of Alzheimer’s Disease Assessment Scale-Cognitive subscale
ANOVA: Analysis of variance
APOE: Apolipoprotein E
AUC: Area under the receiver operating characteristic curve
Aβ: Amyloid-beta
CBS: Corticobasal syndrome
CDR: Clinical dementia rating
CSF: Cerebrospinal fluid
FAQ: Functional activity questionnaire
FBB: Florbetaben
FTLD: Frontotemporal lobar degeneration
GDS: Geriatric depression scale
GM: Gray matter
HC: Healthy controls
IC: Independent component
ICA: Independent component analysis
JART: Japanese adult reading test
LM I: Logical memory immediate recall
LM II: Logical memory delayed recall
MCI: Mild cognitive impairment
MMSE: Mini-mental state examination
MRI: Magnetic resonance images
MTL: Medial temporal lobe
PCR: Polymerase chain reaction
PET: Positron emission tomography
PSP: Progressive supranuclear palsy
ROI: Region-of-interest
SBM: Source-based morphometry
SD: Standard deviation
SHAP: SHapley Additive exPlanations
SVM: Support vector machine
TMT: Trail making test
WF: Word fluency
WM: White matter
