# Supplementary material for: Amyloid-β prediction machine learning model using source-based morphometry across neurocognitive disorders
Source: Sci Rep. 2024 Apr 1;14:7633. doi: 10.1038/s41598-024-58223-3 (PMC10984960; doi:10.1038/s41598-024-58223-3)
Supplement: Supplementary file 1 — Supplementary Legends. [file 41598_2024_58223_MOESM1_ESM.docx]

**Additional Material**

**Additional Figure 1. Flow of Machine Learning**

**Legend:** Structural Brain images were first segmented into gray matter (GM). The preprocessed GM images on which independent component analysis (ICA) was performed for all scans. In the ICA formula, each β represents the weighting coefficient associated with the effect of each independent component (IC) for the GM image and K indicates the number of extracted ICs. We built predictive models for Aβ-positivity. The input feature values were based on the ICA’s β-values, demographic characteristics (i.e., age and sex), cognitive assessments, and apolipoprotein E genotype. Throughout the model building, we used a Gaussian kernel support vector machine (SVM) as the classifier and the model was validated using 5-fold cross-validation. For a 5-fold training/test split, the model was fitted to the training data, and the predictive value was assessed using the test data over all splits (five times). We tuned the hyperparameters (i.e., Gamma and C in SVM and the number of ICs) with a grid search in all model buildings.

**Additional Figure 2. The gray matter volume pattern of each independent component in a three-dimensional brain map derived from source-based morphometry**

**Legend:** Each Figure shows a three-dimensional brain map of independent components 1 (a), 2 (b), 3 (c), 4 (d), 5 (e), 6 (f), and 7 (g). The 3D image was generated using BrainNet Viewer 1.7 (https://www.nitrc.org/projects/bnv).

**Additional Figure 3. Distribution of Aβ-positivity and Aβ-negativity in IC 1 and IC 4, and association with age**

**Legend:** A linear correlation was observed between age and IC 4 (b) but not between age and IC 1 (a) after multiple comparison correction. Orange dots represent Aβ-positivity, blue dots represent Aβ-negativity.

**Abbreviation:** Aβ, Amyloid-β; IC, Independent component.
