# Supplementary figures and images for: Amyloid-β prediction machine learning model using source-based morphometry across neurocognitive disorders
Source: Sci Rep. 2024 Apr 1;14:7633. doi: 10.1038/s41598-024-58223-3 (PMC10984960; doi:10.1038/s41598-024-58223-3)

**
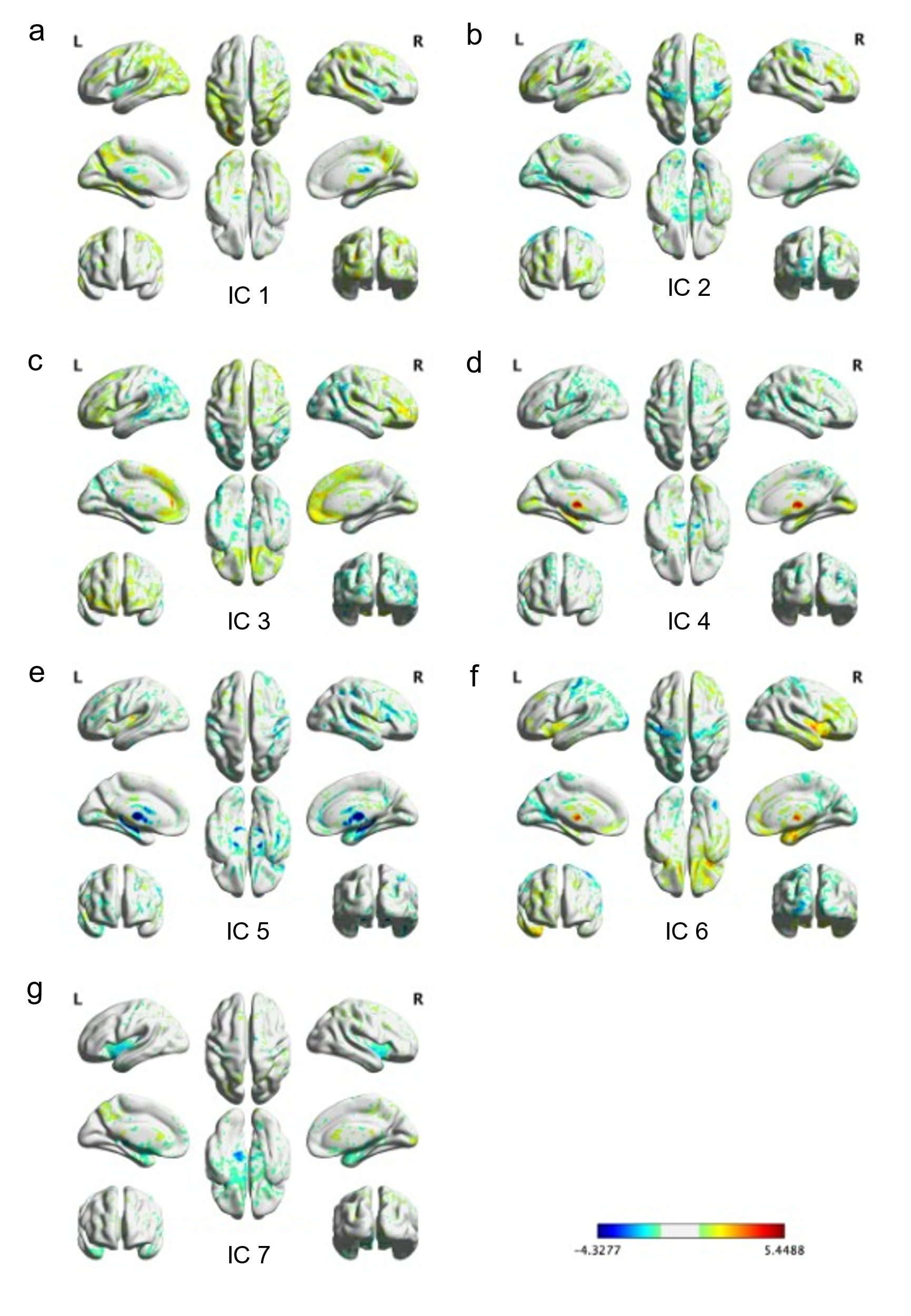
Additional Figure 2**

Supplement: Supplementary file 3 — Supplementary Figure 2. [file 41598_2024_58223_MOESM3_ESM.docx]

**Additional Figure 3**


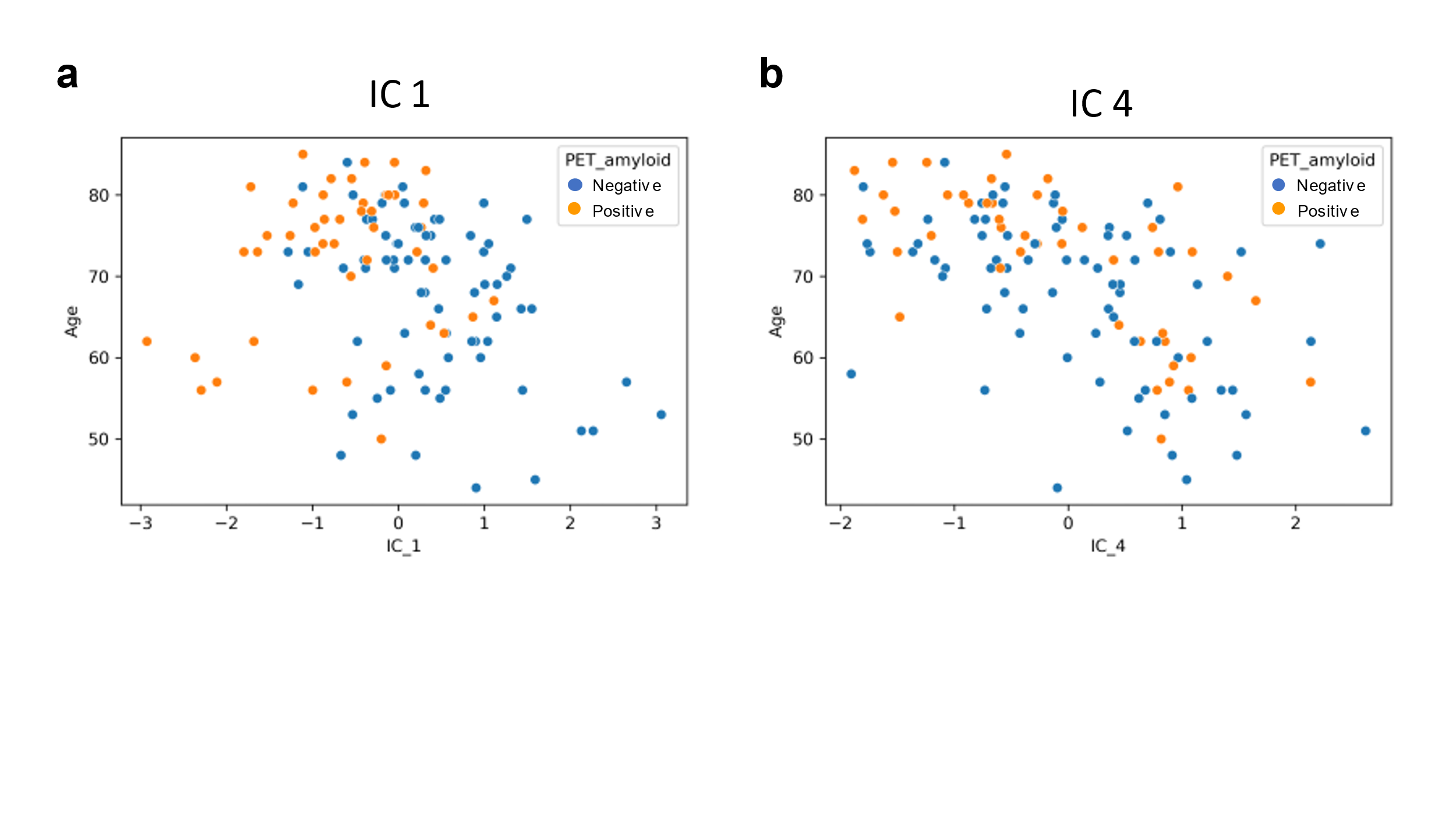

Supplement: Supplementary file 4 — Supplementary Figure 3. [file 41598_2024_58223_MOESM4_ESM.docx]
